# Supplementary material for: Ultralong phosphorescence cellulose with excellent anti-bacterial, water-resistant and ease-to-process performance
Source: Nat Commun. 2022 Mar 2;13:1117. doi: 10.1038/s41467-022-28759-x (PMC8891296; doi:10.1038/s41467-022-28759-x)
Supplement: Supplementary file 1 — Supplementary Information [file 41467_2022_28759_MOESM1_ESM.pdf]

Supplementary Information

Ultralong Phosphorescence Cellulose with Excellent Anti-bacterial, Water-resistant and Ease-to-process Performance

Zhang *et al.*

* Corresponding authors.
E-mail address: zhjm@iccas.ac.cn (J.M. Zhang); jzhang@iccas.ac.cn (J. Zhang).
Mailing address: Zhongguancun North First Street 2,100190 Beijing, PR China

Supplementary Figures

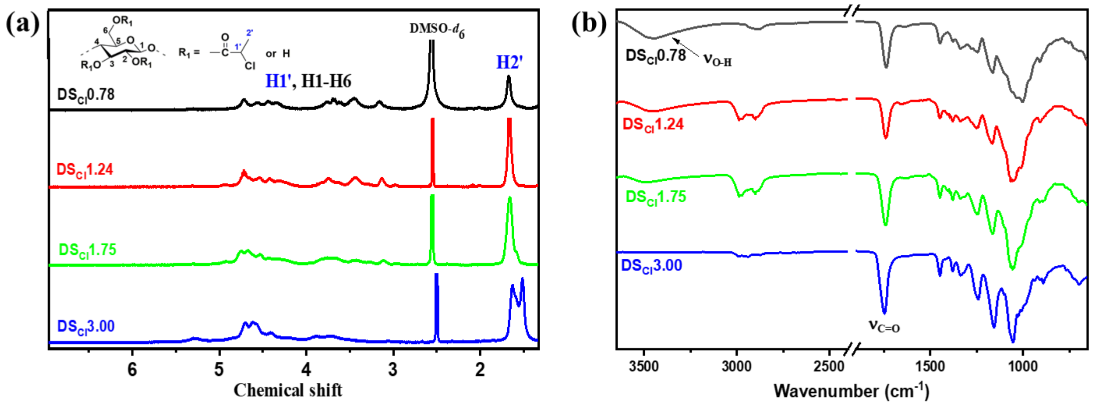

Supplementary Figure 1 (a) ^{1}H-NMR spectra and (b) FTIR spectra of Cell-Cl with different DS_{Cl}.

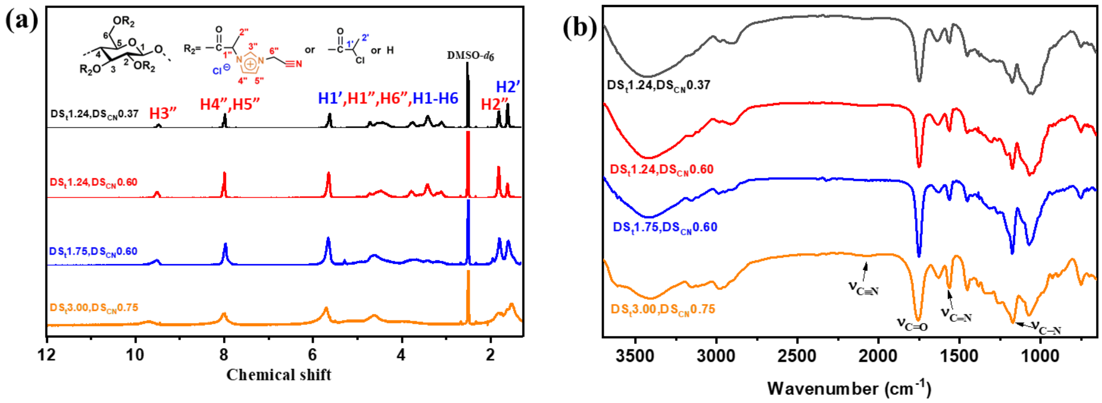

Supplementary Figure 2 (a) ^{1}H-NMR spectra and (b) FTIR spectra of Cell-ImCNCl with different DS_t and DS_{CN}.

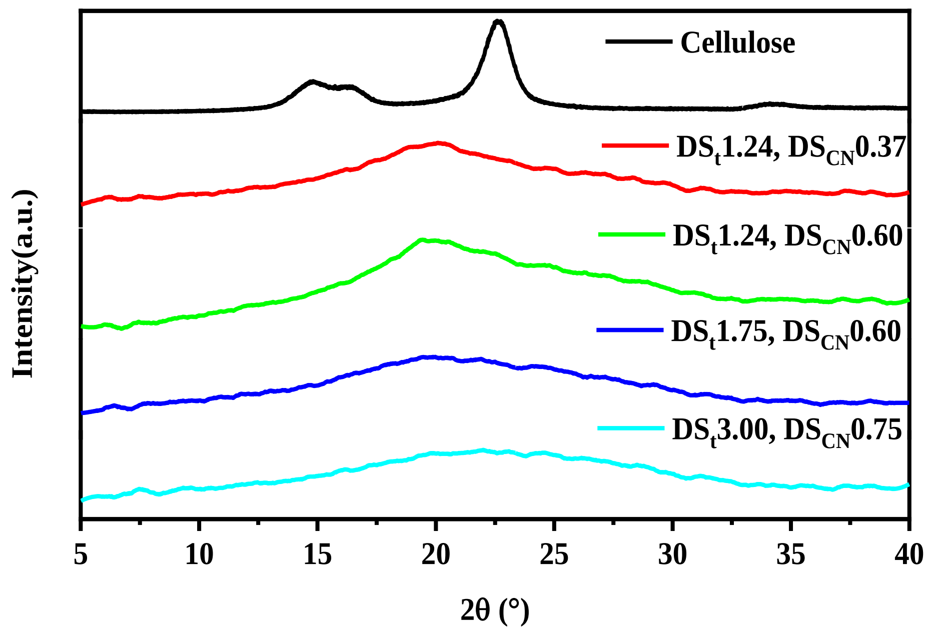

Supplementary Figure 3 XRD curves of cellulose and Cell-ImCNCl with different DS_t and DS_{CN}.

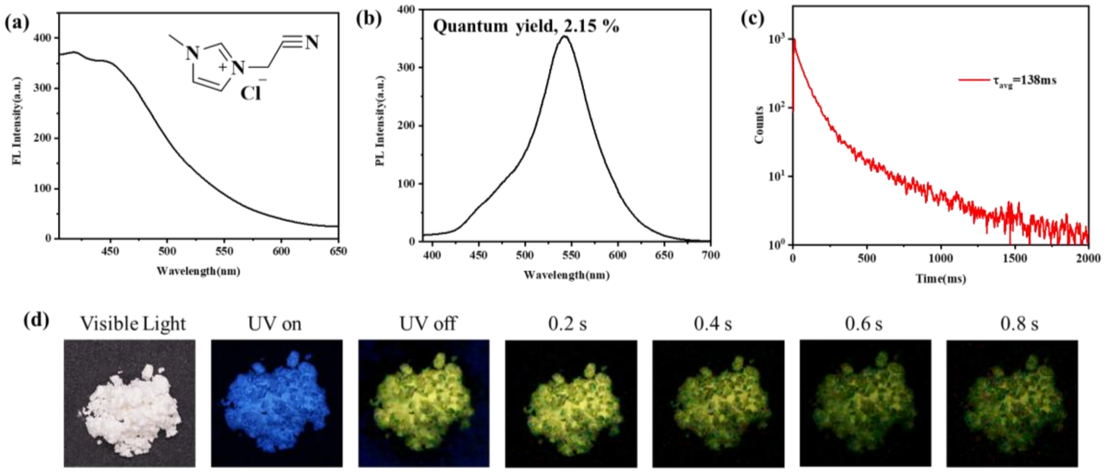

Supplementary Figure 4 (a) Fluorophore spectra of CNMImCl (Ex = 365 nm); (b) RTP spectra of CNMImCl (Ex = 365 nm); (c) RTP lifetime spectra of CNMImCl; (d) Photographs of CNMImCl taken under 365 nm lamp and with the lamp off.

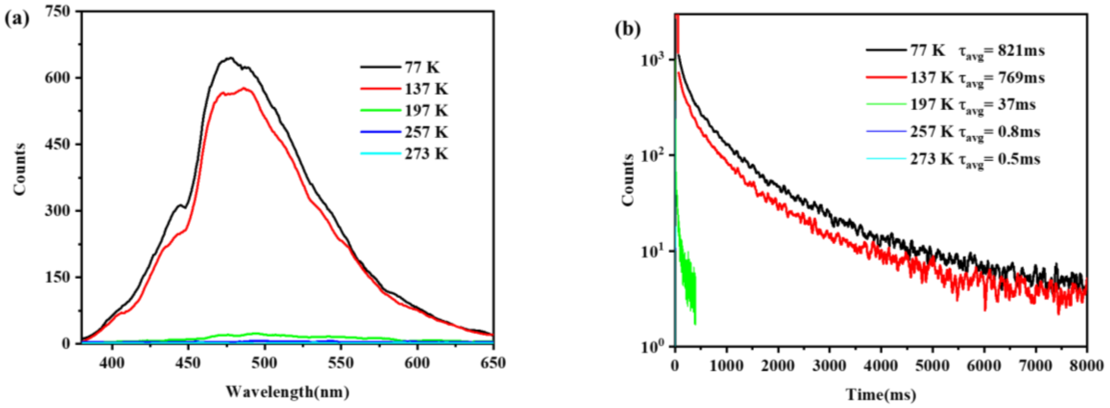

Supplementary Figure 5 (a) RTP spectra of CNMImCl aqueous solution with a concentration of 1 mg/mL at different temperatures; (b) RTP lifetime spectra of CNMImCl aqueous solution with a concentration of 1 mg/mL at different temperatures. (Ex = 365 nm)

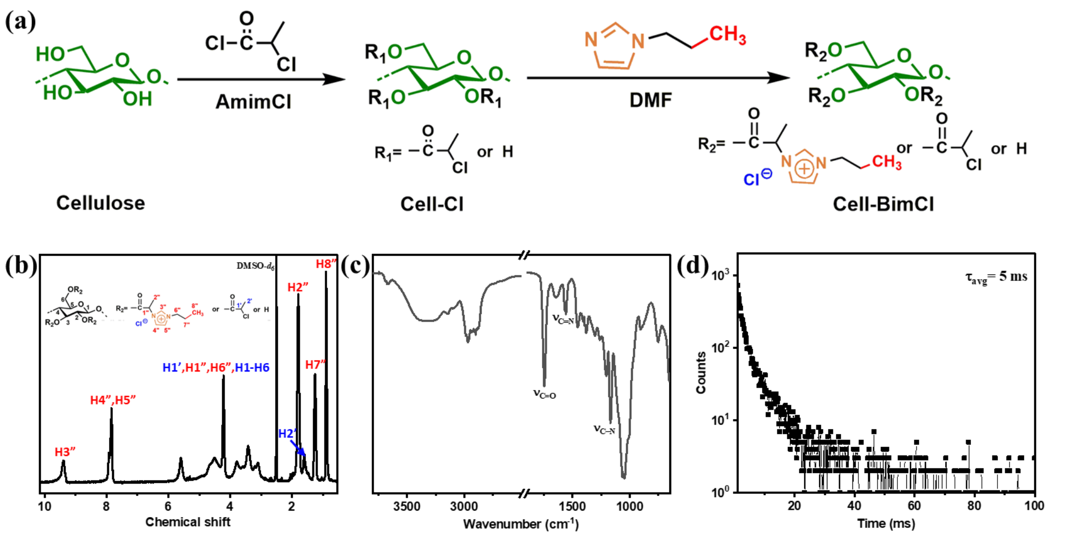

Supplementary Figure 6 (a) Synthesis route of Cell-BimCl; (b) ^{1}H-NMR spectrum of Cell-BimCl; (c) FTIR spectrum of Cell-BimCl; (d) Phosphorescence lifetime spectrum of Cell-BimCl.

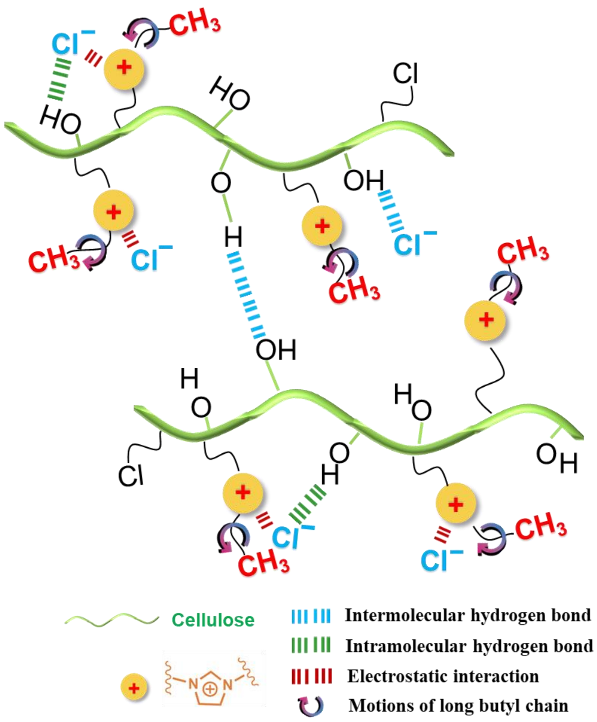

Supplementary Figure 7 Schematic diagram of the aggregate structure of Cell-BimCl.

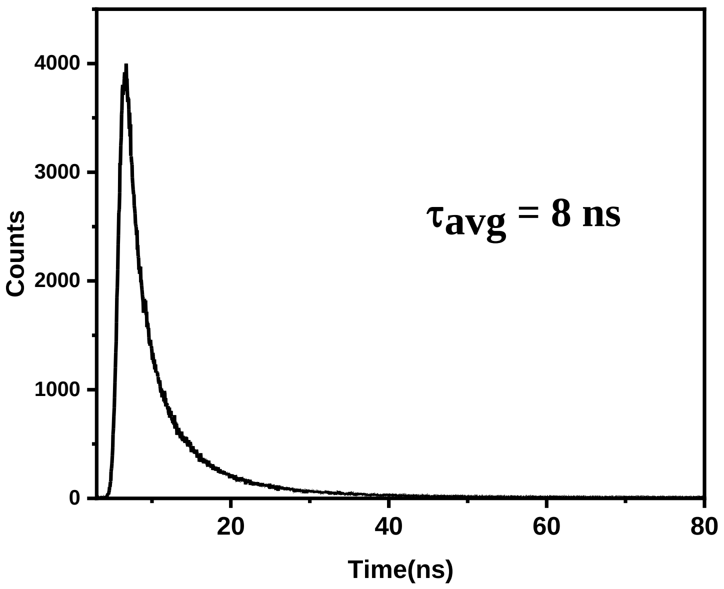

Supplementary Figure 8 Fluorescence lifetime spectrum of 1-cyanomethylimidazole.

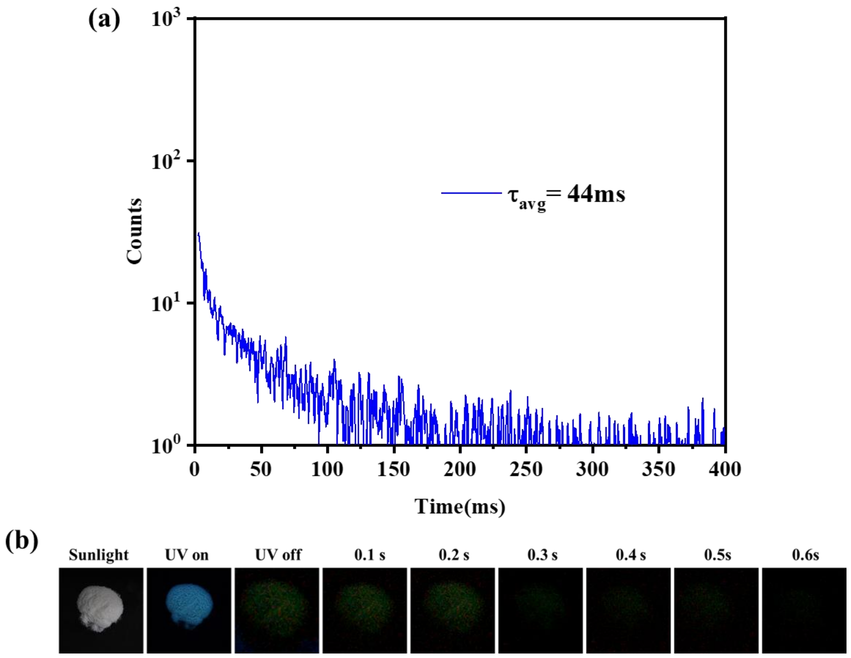

Supplementary Figure 9 (a) RTP lifetime spectra of cellulose powder (Ex = 350 nm); (b) Photographs of cellulose powder taken under 365 nm lamp and with the lamp off.

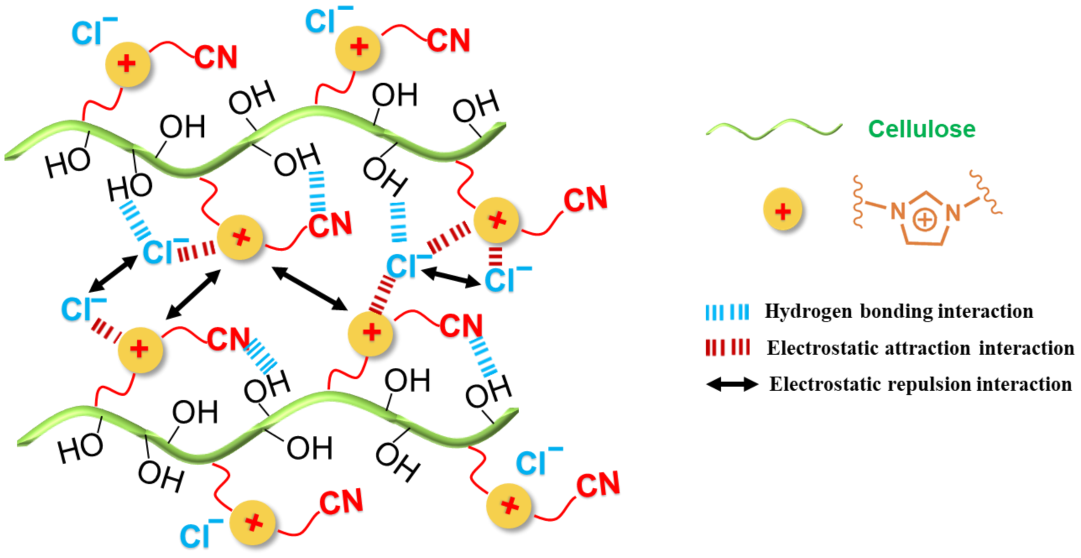

Supplementary Figure 10 Schematic diagram of the aggregation state of Cell-ImCNCl with high DS_{CN}.

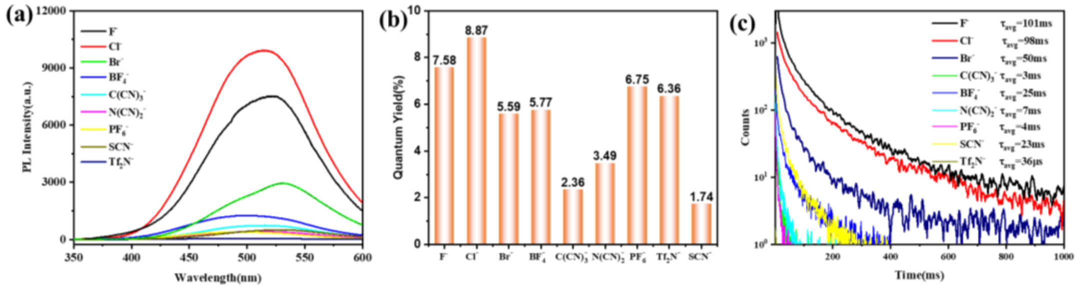

Supplementary Figure 11 (a) RTP spectra of Cell-ImCNX (Ex = 320 nm). (b) Photoluminescence quantum yield of Cell-ImCNX with different anions. (c) RTP lifetime spectra of Cell-ImCNX with different anions.

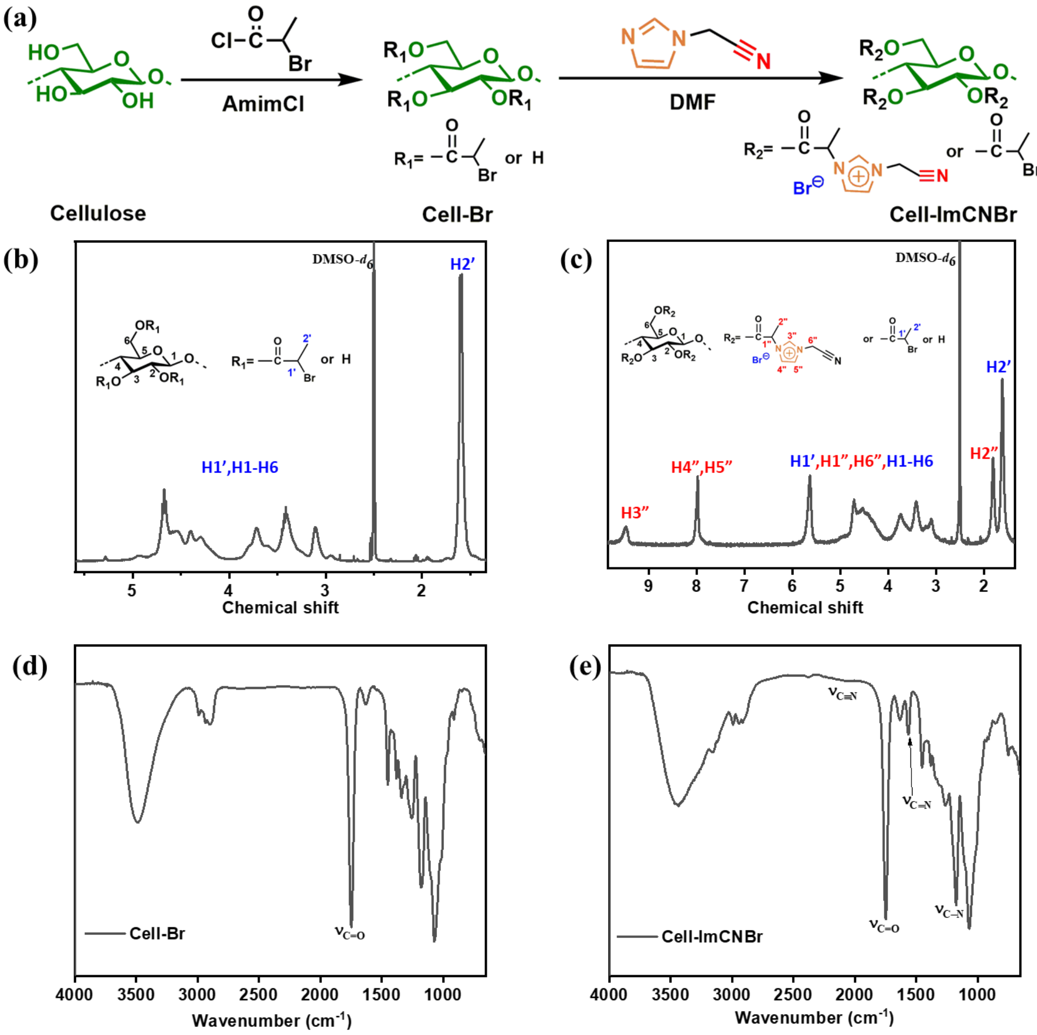

Supplementary Figure 12 (a) Synthetic route of Cell-ImCNBr; (b) ^{1}H-NMR spectrum of Cell-Br; (c) ^{1}H-NMR spectrum of Cell-ImCNBr; (d) FTIR spectrum of Cell-Br; (e) FTIR spectrum of Cell-

ImCNBr.

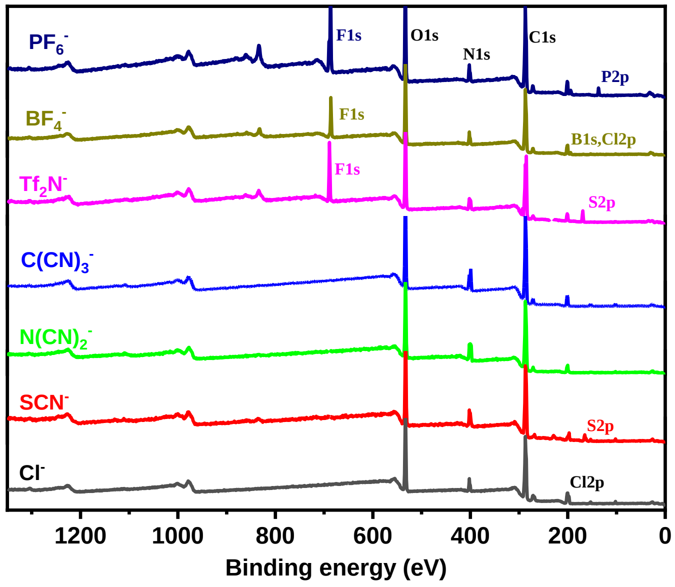

Supplementary Figure 13 XPS curves of Cell-ImCNX.

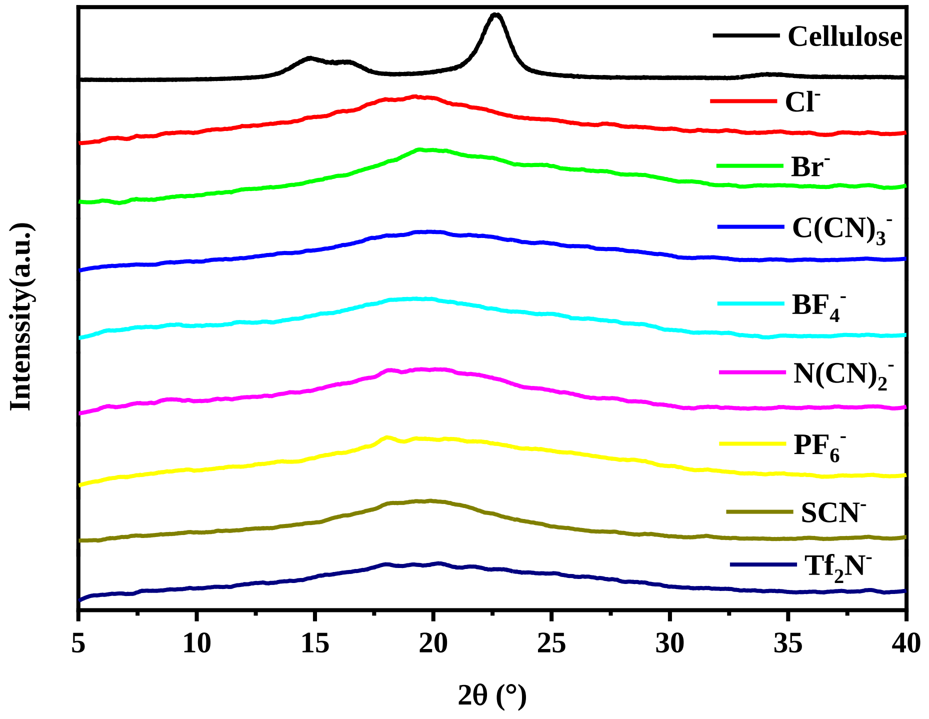

Supplementary Figure 14 XRD curves of cellulose and Cell-ImCNX.

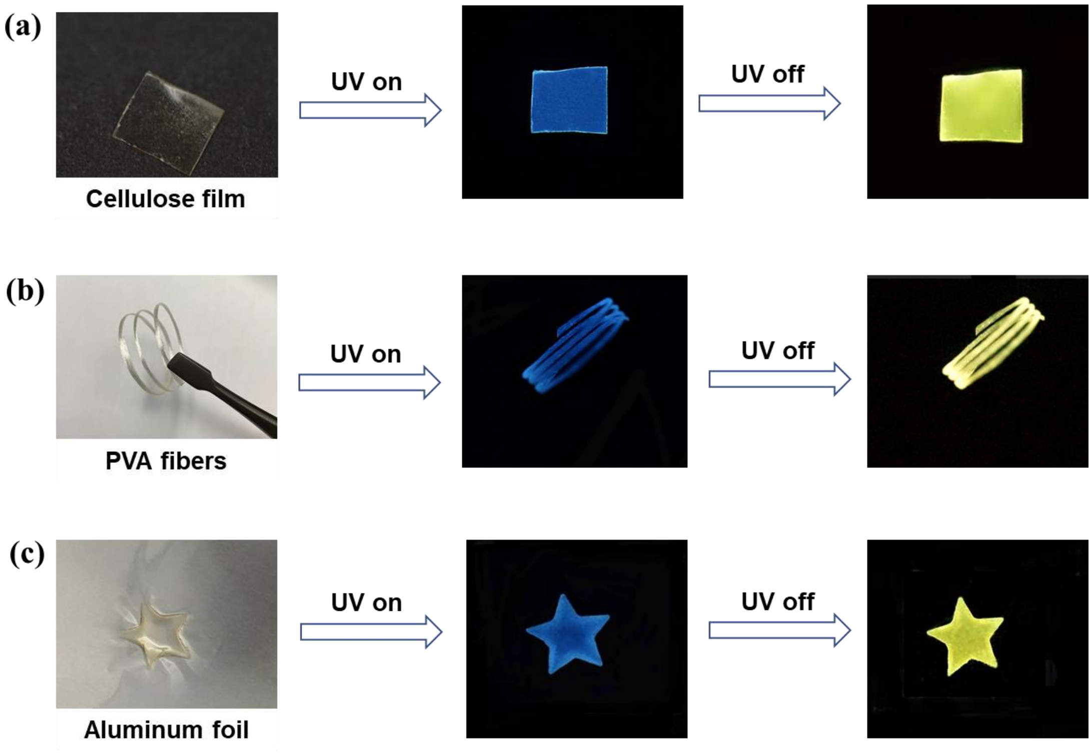

Supplementary Figure 15 (a) Phosphorescent cellulose film prepared by a dip coating method; (b) Phosphorescent PVA fibers obtained by a dip coating method; (c) Phosphorescent pattern prepared on aluminum foil by a mask casting method.

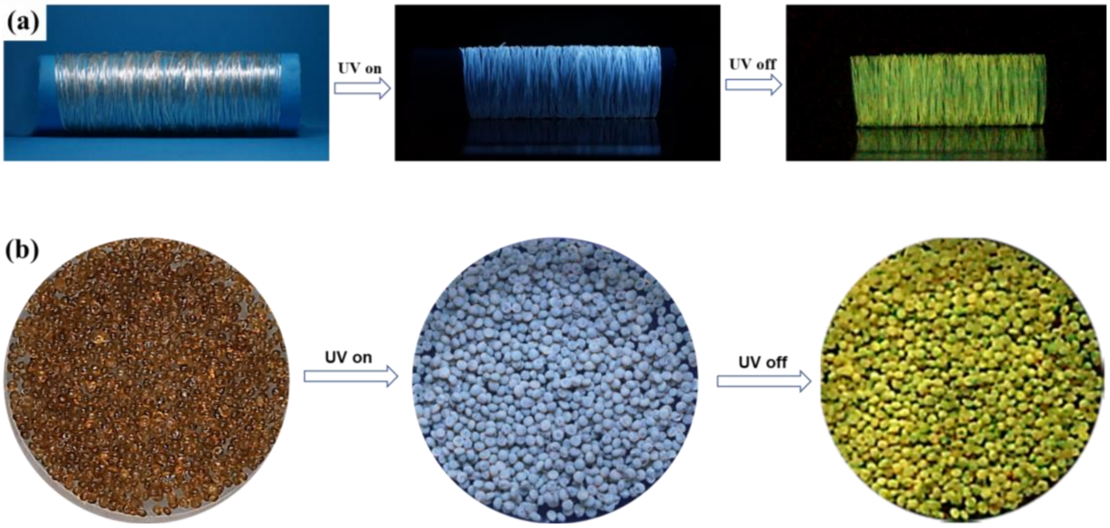

Supplementary Figure 16 (a) The large roll of phosphorescence cellulose fibers. (b) Phosphorescence cellulose microspheres. (Ex = 365 nm)

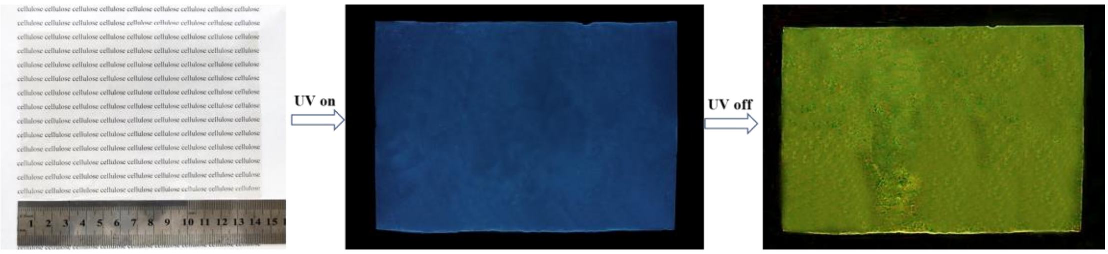

Supplementary Figure 17 The large-ares phosphorescence cellulose film. (Ex = 365 nm)
